# Plant Leaf Detection and Counting in a Greenhouse during Day and Nighttime Using a Raspberry Pi NoIR Camera

**DOI:** 10.3390/s21196659

**Published:** 2021-10-07

**Authors:** Aryuanto Soetedjo, Evy Hendriarianti

**Affiliations:** 1 Department of Electrical Engineering, National Institute of Technology (ITN), Malang 65145, East Java, Indonesia; 2 Department of Environmental Engineering, National Institute of Technology (ITN), Malang 65145, East Java, Indonesia; evyhendriarianti@lecturer.itn.ac.id

**Keywords:** leaf detection, leaf counting, infrared camera, greenhouse, Raspberry Pi

## Abstract

A non-destructive method using machine vision is an effective way to monitor plant growth. However, due to the lighting changes and complicated backgrounds in outdoor environments, this becomes a challenging task. In this paper, a low-cost camera system using an NoIR (no infrared filter) camera and a Raspberry Pi module is employed to detect and count the leaves of *Ramie* plants in a greenhouse. An infrared camera captures the images of leaves during the day and nighttime for a precise evaluation. The infrared images allow Otsu thresholding to be used for efficient leaf detection. A combination of numbers of thresholds is introduced to increase the detection performance. Two approaches, consisting of static images and image sequence methods are proposed. A watershed algorithm is then employed to separate the leaves of a plant. The experimental results show that the proposed leaf detection using static images achieves high recall, precision, and F1 score of 0.9310, 0.9053, and 0.9167, respectively, with an execution time of 551 ms. The strategy of using sequences of images increases the performances to 0.9619, 0.9505, and 0.9530, respectively, with an execution time of 516.30 ms. The proposed leaf counting achieves a difference in count (DiC) and absolute DiC (ABS_DiC) of 2.02 and 2.23, respectively, with an execution time of 545.41 ms. Moreover, the proposed method is evaluated using the benchmark image datasets, and shows that the foreground–background dice (FBD), DiC, and ABS_DIC are all within the average values of the existing techniques. The results suggest that the proposed system provides a promising method for real-time implementation.

## 1. Introduction

Plant growth monitoring is an essential task in agriculture. Compared to the traditional method, which requires direct measurement and is time-consuming, the non-destructive method of using a camera system is an important and challenging topic [1]. Since we may analyze plant growth based on the leaf width, length, and area, as well as the number of leaves, the leaf is the most common part of a plant to be monitored. Moreover, the leaf color may provide information on plant health through the vegetation index [2]. The leaf area and the height of a lettuce plant can be measured using an RGB camera [3], a Kinect sensor [4], a stereo vision system [5], or an NoIR camera [6].

In the leaf monitoring systems discussed previously, leaf parameter measurement and counting are usually performed after the leaf detection or segmentation stage, where the leaves are extracted from the background. It is well known that leaf detection performance relies on environmental conditions, such as the lighting conditions and the complex backgrounds. In this paper, we address leaf detection and counting. Table 1 provides a summary of the existing works in leaf detection and counting; the table lists the algorithms, the hardware and software implementation, the image type, the lighting condition of the image, and the purpose of the algorithm (LD = leaf detection, LC = leaf counting).

As shown in Table 1, there are two approaches for detecting or segmenting a leaf from the background: based on image segmentation techniques [3,4,5,6,7,8,9,10,11,12,13,14,15,16,17,18], and based on machine learning techniques [19,20,21,22,23,24,25,26]. In the first approach, image processing techniques such as color thresholding, superpixel (SLIC), GrabCut [27], watershed, and random walker [28] are commonly employed. Several color spaces such as CIELAB [3,15], HSI/HSV [4,14], and modified red and blue components of RGB [6] are adopted to deal with illumination changes in the color thresholding techniques.

The GrabCut algorithm was employed in [5,16] to detect and measure the area, width, and length of a leaf [5]. Since GrabCut requires a precision marker for better segmentation results, the work in [16] developed markers using the information from the intensity and texture of the image. In [12,17,18], the watershed algorithm was employed to segment occluded cotton leaves. The markers were created using morphological techniques to improve the segmentation results. The superpixel technique using a simple linear iterative clustering (SLIC) was employed in [12] for the leaf segmentation. The superpixel was calculated from the Lab color space; then, the leaf was extracted by simple thresholding of the mean color of the superpixel.

The random walker technique was employed for leaf segmentation in [7,13]. In [7], an interactive tool for annotating the leaf was developed using the random walker to propagate the seed provided by the user. The tool provides an easy method for preparing the leaf dataset. In [13], a robust random walker was proposed to deal with the different illumination images by addressing the illumination of local and nonlocal pixels.

In the second approach, deep neural network (DNN) techniques are commonly employed. The mask region-based convolutional neural network (Mask R-CNN) was employed in [20] for leaf segmentation with a complicated background. More than 4000 leaf images of 15 species were used for the training and testing. The DNNs used for leaf counting were proposed in [22,23,24,26]. In [23], a DNN was employed to count rosette-shaped leaves; this work presented a multimodal leaf counter in which the RGB, near-infrared (NIR), and fluorescent images were taken into account. The dataset contained 2160 images, of which 576 images were annotated. The combination of multimodal images decreased the misclassification error by 50% [23]. 

A Tiny-YOLOv3 network was employed in [24] for real-time leaf counting. The network was trained using 1000 images of *Arabidopsis* plants taken with a Canon camera. The training process was performed on an Intel Core i7 PC for ~2.5 days. The inference time of the Tiny-YOLOv3 implemented on an Intel Core i7 PC was ~10 ms, and was ~5 s on an Android device. The authors in [26] proposed preliminary work for the leaf counting of uncontrolled images in a greenhouse. The system captured the leaves with a smartphone and sent them to the cloud to count the leaves using the DNN.

As discussed previously, leaf segmentation and counting using image processing techniques offers a simple implementation, as it requires neither a large dataset for the training process nor special computer hardware, which are common burdens of machine learning techniques. However, machine learning techniques provide higher performance in the detection and counting, as reported in [20], where the misclassification error of the DNN was the lowest compared to the GrabCut and Otsu thresholding techniques.

As listed in Table 1, most of the existing works implement the algorithms on a PC; thus, this approach is not suitable for real-time monitoring. Only a few works have implemented algorithms on an embedded platform, such as using a Raspberry Pi [6] and an Android phone [24]. Furthermore, leaf images are usually taken during the daytime over a long time interval; for example, every 20 min and 2 h (during daytime) [11], 15 images per day (from 9 a.m. to 11 p.m.) [23], twice per day [24], and 14 images per day (from 9 a.m. to 3 p.m.) [26]. 

The most challenging problems of real-time monitoring in outdoor environments are the illumination changes and the shadows. To deal with these issues, we need image data taken continuously from the environments in order to evaluate the algorithms better. This paper proposes a real-time system for detecting and counting leaves in the daytime and nighttime (24 h continuously) in an outdoor environment, where the image datasets are taken every 10 minutes over the course of six days. 

The main contributions of our work are as follows: First, it employs a low-cost infrared camera (NoIR: no infrared filter camera) to capture the leaf images during the daytime and nighttime in the outdoor environment. Second, it employs an embedded device (Raspberry Pi module) as the image processor, which is easy to install on-site for leaf detection and counting. Third, the proposed approach combines bi-level and multilevel Otsu thresholding techniques for efficient leaf segmentation. Fourth, the proposed approach employs the temporal data images (image sequences) for a better performance. Fifth, the proposed system provides an efficient algorithm for leaf detection and counting without a training process, as well as the image dataset; the latter is obtained by exploiting the image processing techniques and taking advantage of the infrared images captured by the NoIR camera. Compared to the existing methods, our method offers simple algorithms and low-cost devices. Still, it is adequate to handle natural outdoor conditions such as a lack of light at nighttime, illumination changes, and shadow problems during the daytime.

This paper is structured as follows: The proposed algorithms are described in Section 2. Section 3 discusses the experimental results. The conclusion is presented in Section 4.

## 2. Proposed Algorithm

### 2.1. Image Acquisition

**Figure 1:**
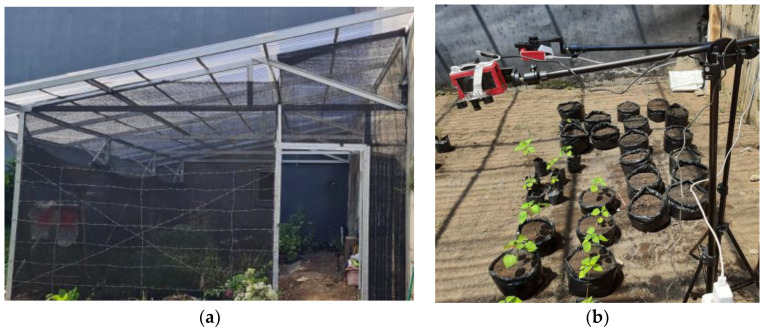
(**a**) Greenhouse; (**b**) *Ramie* plant and camera installation.

In this work, we investigated the leaf detection of the *Ramie* (*Boehmeria nivea Gaud*.) plant, which is used in the phytoremediation process to eliminate pollutants in wastewater using a green plant. The image data of the *Ramie* leaves were collected using an infrared camera installed at a greenhouse. The greenhouse was covered with a transparent roof, while the right and back sides were concrete walls and the left and front sides were covered with a shaded net, as illustrated in Figure 1a.

One week old *Ramie* plants were planted in poly bags and placed in the greenhouse. At this age, the *Ramie* leaves are separated, and they can be counted when the images are taken from the top. Therefore a Raspberry Pi NoIR camera was installed on a tripod and captured the plants from the top-side view. The plant and camera arrangement is shown in Figure 1b. There was no artificial light in the greenhouse; thus, the sunlight was the lighting source during the daytime, and it was dark at night.

**Figure 2:**
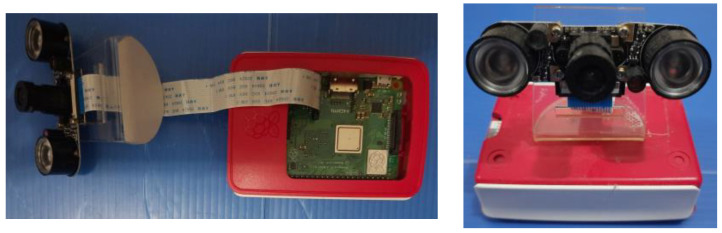
Raspberry Pi NoIR camera module.

Figure 2 depicts the Raspberry Pi NoIR camera; it consisted of a Raspberry Pi 3 Model B+, a 5 megapixel Omnivision 5647 camera module (without an infrared filter), and a pair of infrared LEDs. Since there was no infrared filter on the camera module, it was sensitive to the infrared light (around 880 nm). It is noted here that the NoIR camera provided normal RGB data; thus, the image captured by the module was the standard RGB image. The camera was connected to the Raspberry Pi module using a camera serial interface (CSI) that provided a high data rate. A MotionEyeOS [29] was installed on the Raspberry Pi for image capture. The Raspberry Pi camera system ran continuously from 24 April to 29 April 2021 for image collection. The images were captured every 10 minutes and uploaded to Google Drive. There were a total of 758 images stored in Google Drive for the evaluation. Over the course of six days, the camera arrangements were not changed abruptly. However, the camera was slightly aligned a few times due to the wind or other physical disturbances. 

**Figure 3:**
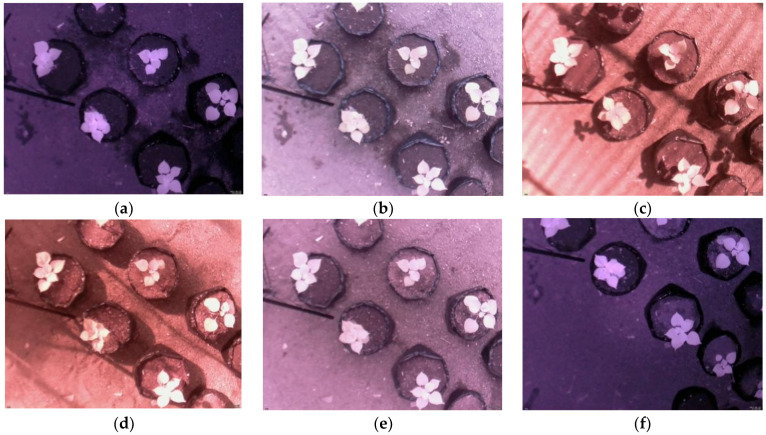
Images of *Ramie* plants taken at different times: (**a**) 03:00; (**b**) 07:00; (**c**) 10:00; (**d**) 14:30; (**e**) 16:50; (**f**) 21:00.

The samples of images captured by the NoIR camera are depicted in Figure 3, where Figure 3a–f show the images taken at 03:00, 07:00, 10:00, 14:30, 16:50, and 21:00, respectively. From the figures, several facts can be ascertained, as follows:

- A low-cost Raspberry Pi NoIR camera can capture leaves in natural outdoor environments during the day and nighttime;
- The image intensity frequently changes according to the time of day;
- The colors of backgrounds (non-leaf) vary according to the lighting;
- The shadow problem occurs during the daytime (Figure 3c,d);
- The color of the leaves is more uniform during the nighttime (Figure 3a,f), but it appears non-uniform during the daytime (Figure 3b–e);
- Strong sunlight causes the color of the soil to become a white color, similar to the leaf color.

The abovementioned facts led us to develop an algorithm to extract the leaves from the background and count the number of leaves, as described in the next section.

### 2.2. Overview of Proposed Algorithm

The main objective of our work was to develop an efficient algorithm for leaf detection and counting that could be implemented on a low-cost embedded system (Raspberry Pi module) for real-time monitoring in the outdoor environment. The leaf detection extracted the bounding boxes of detected leaves from the backgrounds. Since each *Ramie* plant was planted in a poly bag and arranged separately, the bounding box was used to detect each plant in the image. Once the bounding box was detected, the leaf counting algorithm was applied to count the number of leaves on each plant. 

**Figure 4:**
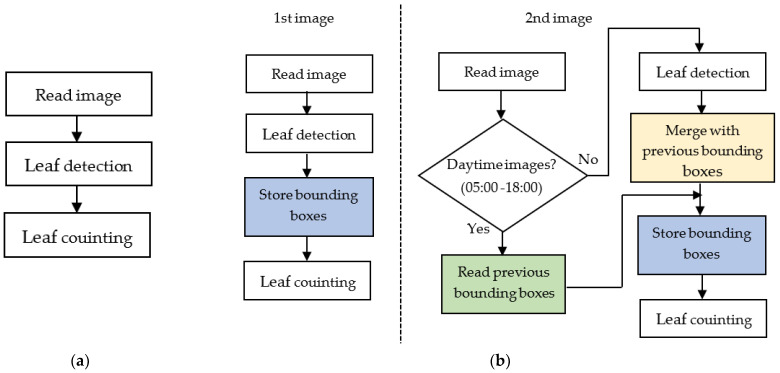
Flowchart of proposed systems: (**a**) static image approach; (**b**) image sequence approach.

The flowchart of the proposed system is depicted in Figure 4, where the method in Figure 4a is a static image approach, while the method in Figure 4b is the image sequence approach utilizing the temporal images. In the static image approach, the images are treated independently, where after reading an image, leaf detection and counting are performed. The image sequence approach exploits the temporal data of images; it takes advantage of both the sequence of images and the timestamps of the images. To provide an easy explanation, the first image in Figure 4b is assumed to be the first image in the sequence taken by the camera.

The idea to utilize a sequence of images was based on the observation that some of the leaves were not detected in several images. Therefore, incorporating the information of detected leaves in the previous pictures may solve this problem. Meanwhile, the timestamps of the images were used to identify the day or night images. Based on the observations in Figure 3, the leaves were difficult to detect due to the shadow problems. Thus, using the previously detected leaves in the night images is better than performing standard leaf detection. However, these conditions do not always hold, in the sense that in some cases, the first image sequence was the day image. In such a situation, we should adopt standard leaf detection.

As depicted in Figure 4b, the algorithm in the first image introduced a process to store the detected bounding boxes after the leaf detection. In the second image, the algorithm checked whether the image was the day image or night image. If the image is the day image (from 05:00 to 18:00), the algorithm will read the previously stored bounding boxes. The algorithm will merge the previously stored bounding boxes with the current detection if the image is the night image.

### 2.3. Leaf Detection and Counting

Our proposed leaf detection algorithm was based on the observation of the images shown in Figure 3. The observation of the images suggests that the infrared images provided a better leaf segmentation. We could extract the leaves from the backgrounds using a simple thresholding technique. However, each image required a different number of thresholds for proper segmentation. Let us examine a grayscale image, where its intensity (*Y*) is obtained from the *R, G*, and *B* components using the weighted sum, as follows [30]:

(1) Y=0.299R+0.587G+0.114B. 

**Figure 5:**
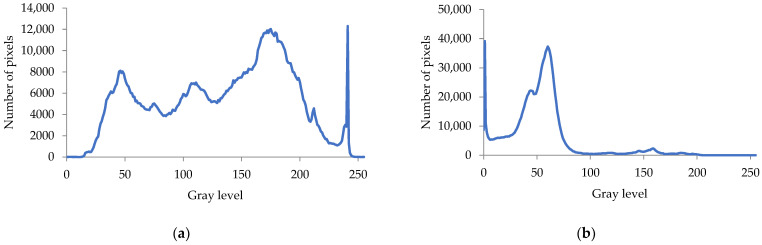
(**a**) Histogram of image in Figure 3c. (**b**) Histogram of image in Figure 3f.

Figure 5a,b illustrate the histograms of the grayscale images in Figure 3c,f, respectively. Figure 5a shows several peaks in the histogram; thus, it was necessary to adopt multilevel thresholding to extract the foreground (leaf). In contrast, Figure 5b shows only two peaks; thus, bi-level thresholding was appropriate. Therefore, we developed an approach to combine bi-level and multilevel thresholding for effective leaf segmentation.

**Figure 6:**
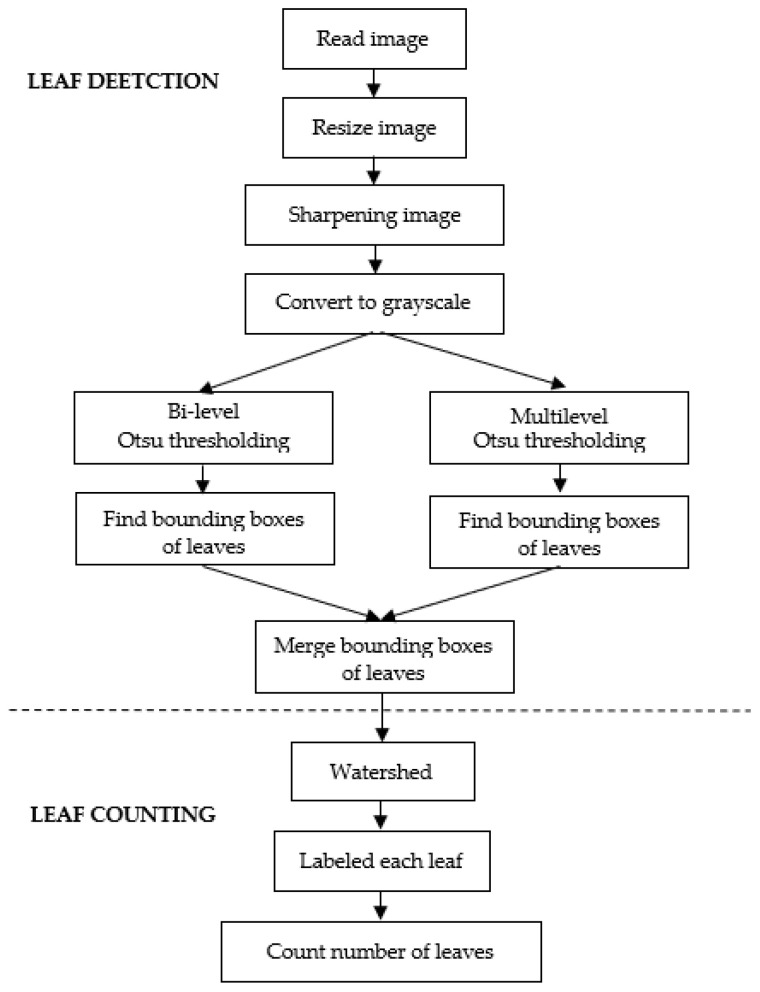
Flowchart of the proposed leaf detection and counting method.

The flowchart of the proposed leaf detection and counting method is depicted in Figure 6. It started with the reading of an RGB image. The image size collected from the greenhouse was 1280 × 1024 pixels. The image was resized to 640 × 512 pixels to speed up the process. Then, the image was sharpened using a sharpening filter to enhance the contrast between the objects. The sharpening filter was a spatial filter using a 3 × 3 Laplacian kernel (all elements of the kernel were −1, except for the center one, which was 9). Recalling the grayscale histograms in Figure 5, it was clear that the grayscale image of the respective RGB image was suitable for the leaf segmentation. Therefore, after sharpening, the RGB image was converted to a grayscale image.

As described previously, our algorithm combines bi-level and multilevel thresholding to accommodate the day and nighttime images; therefore, both bi-level Otsu thresholding and multilevel Otsu thresholding were applied to the grayscale image. Otsu thresholding is an adaptive image segmentation technique that selects the threshold automatically. Bi-level Otsu thresholding uses a single optimal threshold that maximizes the interclass variance [31]. Multilevel Otsu thresholding is an extension of bi-level Otsu thresholding, where two or more thresholds are employed, as proposed by [32,33]. The algorithm is described in the following text. Assuming that a grayscale image contains *N* pixels with the *L* gray levels (0, 1, …, *L*−1), the number of pixels at the gray level *i* is denoted as *f_i_*; then, the probability of gray level *i* (*p*_i_) is expressed as:

(2) $$p_{i}=\frac{f_{i}}{N}.\;$$

To segment an image into *M* classes (*C*_1_, *C*_2_, …, *C*_M)_), we need *M*−1 thresholds (*Th*_1_, *Th*_2_, …, *Th_(_*_M-1)_). The cumulative probability for each class *C_k_* (*ω_k_*) is expressed as:

(3) $$\omega_{k}=\underset{i\in C_{k}}{\sum }p_{i}.\;$$

and the mean gray level for each class *C_k_* (*μ_k_*) is expressed as:

(4) $$\mu_{k}=\underset{i\in C_{k}}{\sum }i\frac{p_{i}}{\omega_{k}}$$

The mean intensity for a whole image (*μ_T_*) is expressed as:

(5) $$\mu_{T}=\sum_{k=1}^{M}\omega_{k}\mu_{k}.\;$$

and the interclass variance ($\sigma_{B}^{2}$) is expressed as:

(6) $$\sigma_{B}^{2}=\sum_{k=1}^{M}\omega_{k}{\left(\mu_{k}-\mu_{T}\right)}^{2}$$

The optimal thresholds (*Th*_1_ *, *Th*_2_ *, …, *Th*_M-1_ *) can be defined by maximizing the interclass variance as:

(7) $$\left\{Th_{1}^{*},\;Th_{2}^{*},\;\ldots ,\;Th_{M-1}^{*}\right\}=\underset{0\ll Th_{1}<\ldots <L-1}{\arg}max\left\{\sigma_{B}^{2}\left(Th_{1},\;Th_{2},\;\ldots ,\;Th_{M-1}\right)\right\}$$

Therefore, the Otsu thresholding is the iteration method, wherein each step updates the *ω_k_* and *μ_T_* to calculate the interclass variance ($\sigma_{B}^{2}$). Finally, the optimal thresholds are selected when the interclass variance is at its maximum. 

After performing the Otsu thresholding, the bounding boxes of detected leaves were found. Since the bounding boxes may contain non-leaf objects, an additional process was required to discard incorrect bounding boxes. This process discarded the detected objects based on their area and shape similarity. The area of an object was determined by the contour area of the connected components of the object. The shape similarity was calculated using the Hu moment of the image [34]. 

The final step in the leaf detection was to merge the bounding boxes obtained by both the bi-level and multilevel thresholding. When the bounding boxes were generated by the bi-level thresholding but not generated by the multilevel thresholding, or vice versa, these bounding boxes were directly merged into the final list. However, when they were closed to one another, the following rules were employed to merge them: *xa_min* and *xa_max* were the x-coordinates of the left and right positions of the first bounding box; *ya_min* and *ya_max* were the y-coordinates of the top and bottom positions of the first bounding box; *xb_min* and *xb_max* were the x-coordinates of the left and right positions of the second bounding box; *yb_min* and *yb_max* were the y-coordinates of the top and bottom positions of the second bounding box. Both bounding boxes were merged if one of the following conditions was satisfied:

(8) |ya_min−yb_min|<30 AND |xa_min−xb_min|<30 

(9) |ya_min−yb_min|<30 AND |xa_max−xb_max|<30 ,

(10) |ya_max−yb_max|<30 AND |xa_min−xb_min|<30 ,

(11) |ya_max−yb_max|<30 AND |xa_max−xb_max|<30.

When two bounding boxes were merged in to the final list, the bounding box with the higher shape similarity was selected.

Once the bounding boxes were defined, the leaf counting algorithm was applied to count the leaves on each plant (bounding box). A watershed algorithm was employed to separate the leaves of the *Ramie* plant. The watershed algorithm is a popular technique to separate objects that are touching [35]. The idea of the algorithm consists of considering an image as the topographical surface and performing a flooding process in the catchment basins to create a dam, called the watershed. The flooding starts by pouring water into the valley (local minima) until the water fills all of the catchment basins. The barrier (watershed) is then built to prevent the water from the different valleys from merging.

The implementation of the watershed algorithm in the software was as follows [36,37]: The ordered queue was used to simulate the flooding process of the watershed algorithm. The ordered queue consisted of a series of simple queues, where each simple queue represented the gray level of the image. In the event that the gray level varies from 0 to 255, there are 256 queues in a series. The queue that corresponded to the gray level 0 was the highest priority. The element was removed from the queue based on the priority.

The algorithm was composed of the initialization phase, followed by the working phase. The algorithm’s input was an image *f* and a set of markers *M*, where the algorithm will flood the image *f* with the sources from marker *M*. The output of the algorithm was a flooded image *g*.

A. Initialization phase:
  1. Create an ordered queue, where the number of simple queues equals the number of gray levels in an image *f*;
  2. Select all boundary points of the markers and put them into the ordered queue, where the gray value of the point determines its priority in the ordered queue. For instance, the marker with the gray level value of 0 is entered into the highest priority of the ordered queue, while the one with the value of 255 is entered into the lowest priority of the ordered queue.

B. Working phase:
  1. Create an image *g* by labeling the markers *M*;
  2. Scan the ordered queue from the highest priority queue;
  3. Remove an element *x* from the first non-empty ordered queue;
  4. Find each neighbor *y* of *x* in the image *g* that has no label;
  5. Label the point *y* obtained in Step B.4 with the same label of *x*;
  6. Store the point *y* obtained in Step B.4 in the ordered list, where the gray value of point *y* determines its priority in the ordered queue;
  7. If all queues in the ordered queue are empty, stop the algorithm; otherwise, proceed to Step B.2

The standard watershed algorithm described above may produce over-segmentation due to the initial markers, which represent the noise. Therefore, the markers were selected from the known objects (leaves) based on the distance transform of the binary image, as described in the following text. The leaf detection algorithm generated a binary (thresholded) image, where the white color represents the foreground (leaf object) and the black color represents the background. The Euclidean distance transform of the image (*EDT*(*x,y*)) can be calculated as [38]:

(12) $$EDT(xy)=\{\begin{array}{ll} 0 & I\left(x,y\right)\in \left\{Bg\right\}\;\\ min\left(\sqrt{{\left(x-x_{0}\right)}^{2}+{\left(y-y_{0}\right)}^{2}}\right),\;\forall I\left(x_{0},y_{0}\right)\in Bg\; & I\left(x,y\right)\in \left\{Ob\right\} \end{array}$$

where *Bg* and *Ob* are the background and the leaf object, respectively. The maxima of *EDT*(*x,y*) represent the center points of leaves. Thus, they were then selected as markers in the watershed algorithm.

### 2.4. Performance Evaluation

To evaluate the performance of the proposed leaf detection method, we used the following metrics: recall, precision, F1 score, and foreground–background dice (FBD) [11,22]. Recall represents the portion of ground truth leaves that appear in the detected leaves, and can be expressed as:

(13) $$\mathrm{Recall}=\frac{\mathrm{TP}}{\mathrm{TP}+\mathrm{FN}}\;$$

where TP stands for true positive, denoting the detected leaf as a correct detection, while FN stands for false negative, denoting an undetected leaf. Precision represents the portion of detected leaves that match with the ground truth leaves, and can be expressed as:

(14) $$\mathrm{Precision}=\frac{\mathrm{TP}}{\mathrm{TP}+\mathrm{FP}}$$

where FP stands for false positive, denoting a detected leaf as a false detection. The F1 score represents the harmonic mean of recall and precision, and can be expressed as:

(15) $$F1\;\mathrm{score}=\frac{2\times \mathrm{Precision}\times \mathrm{Recall}}{\mathrm{Precision}+\mathrm{Recall}}.\;$$

FBD represents the segmentation accuracy by measuring the overlap area of the segmentation result and the ground truth, and can be expressed as:

(16) $$\mathrm{FBD}=\frac{2\times \left|P_{\mathrm{sg}}\cap^{\;}P_{\mathrm{gt}}\right|}{\left|P_{\mathrm{sg}}\right|+\left|P_{\mathrm{gt}}\right|}$$

where P_sg_ and P_gt_ are the foreground area of the segmentation result and the ground truth, respectively. It is noted here that TP, FP, and FN are calculated based on the bounding box of the leaf, while the P_sg_ and P_gt_ are based on the leaf area. High leaf detection performance is indicated by values of recall, precision, F1 score, and FBD closer to 1.

To evaluate the performance of the proposed leaf counting method, we used the following metrics: difference in count (DiC), and absolute difference in count (ABS_DIC) [11,22]. DiC represents the difference in number between the ground truth and the leaf counting algorithm, and can be expressed as:

(17) $$\mathrm{DiC}=\frac{1}{N}\overset{N}{\underset{i=1}{\sum }}\left(GT_{i}-LF_{i}\right)$$

where GT is the number of ground truth leaves, LF is the number of leaves calculated by the algorithm, and *N* is the number of samples. ABS_DiC is the absolute value of the difference in numbers between the ground truth and the leaf counting algorithm, and can be expressed as:

(18) $$\mathrm{ABS}\_\mathrm{DiC}=\frac{1}{N}\overset{N}{\underset{i=1}{\sum }}\left|{\mathrm{GT}}_{i}-{\mathrm{LF}}_{i}\right|$$

High leaf counting performance was indicated by values of DiC and ABS_DiC closer to 0.

## 3. Experimental Results

In the experiments, the proposed algorithm was implemented on a Raspberry Pi 3 Model B+. The Raspberry Pi employed the Raspberry Pi OS operating system. The program was written using the Python language and the OpenCV library. The metrics described in the previous section, along with the execution time, were used to evaluate the performance. The objective was to assess the reliability of the proposed system for implementation in real time in outdoor natural environments. 

### 3.1. Leaf Detection Results

As described in Section 2.2, our proposed leaf detection method combined bi-level and multilevel Otsu thresholding. The approach consisted of two methods: static image and image sequence methods. In the static image method, six methods—namely, M1 to M6, as described in Table 2—were examined to evaluate the effectiveness of the proposed algorithm. M1 to M3 used one thresholding method (no combination): M1, M2, and M3 used bi-level thresholding (single threshold), three-level thresholding (two thresholds), and four-level thresholding (three thresholds), respectively. M4 to M6 combined two thresholding methods: M4, M5, and M6 combined M1 and M2, M1 and M3, and M2 and M3, respectively. Meanwhile, there were two methods in the image sequence method—namely, M4_SQ (method M4 with the sequence of images) and M5_SQ (method M5 with the sequence of images). Furthermore, the SLIC method proposed by [12] was used for comparison.

#### 3.1.1. Leaf Detection Results Using the NoIR Camera

**Figure 7:**
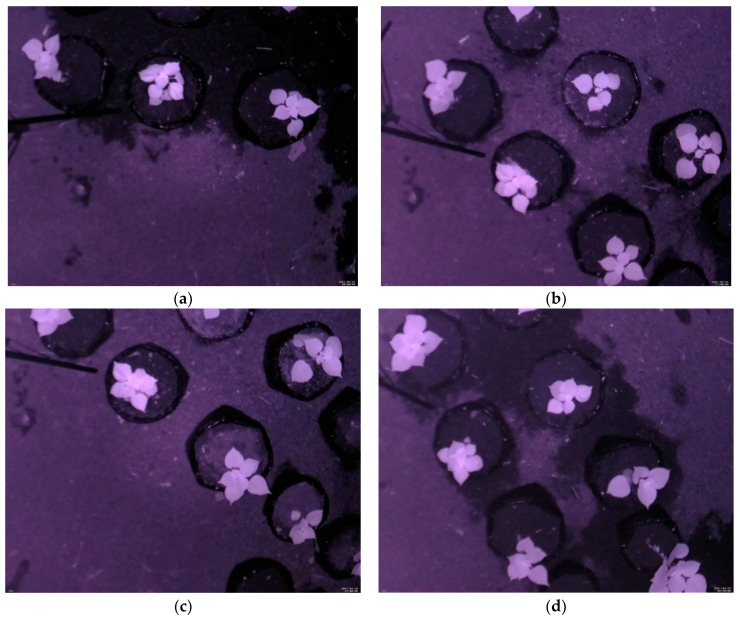
Sample of image data in (**a**) Scene-1; (**b**) Scene-2; (**c**) Scene-3; (**d**) Scene-4.

The proposed leaf detection algorithm was tested using the image data collected from the greenhouse, as described in Section 2.1. There were 758 images divided into 4 scenes (Scene-1 to Scene-4), as illustrated in Figure 7. Scene-1, Scene-2, Scene-3, and Scene-4 contained 47, 301, 115, and 295 images, respectively. Each scene differed in terms of the position and the number of the *Ramie* plants in the image. The number of *Ramie* plants in Scene-1 was three. Scene-2 and Scene-3 had five plants, while Scene-4 had six plants. It is noted here that each scene contained both day and nighttime images. 

**Figure 8:**
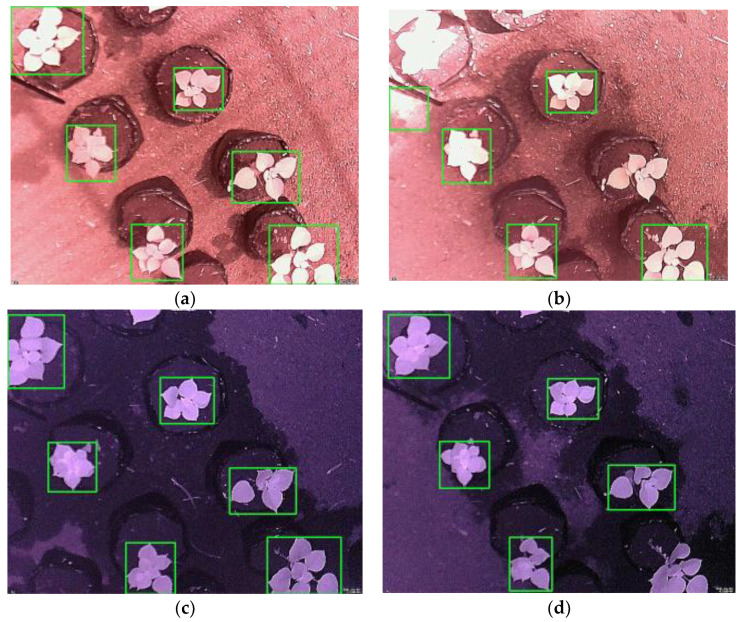
Leaf detection results: (**a**) daytime image with TP = 1, FP = 0, FN = 0; (**b**) daytime image with TP = 0.67, FP = 0.17, FN = 0.33; (**c**) nighttime image with TP = 1, FP = 0, FN = 0; (**d**) nighttime image with TP = 0.83, FP = 0, FN = 0.17.

Typical leaf detection results are illustrated in Figure 8, where Figure 8a,b show the results of the daytime images, while Figure 8c,d show the results of the nighttime images. As described previously, leaf detection was used to find the bounding boxes of the leaves of *Ramie* plants in the poly bags, as shown in the figure. Thus, a detected leaf was represented by a detected plant. The images with a TP of 1, an FP of 0, and an FN of 0 are shown in Figure 8a,c. The proposed method could detect all plants in images with shadows, as shown in Figure 8a. In Figure 8b, two plants were not detected, while one non-leaf object was detected; the TP was 0.67, the FP was 0.17, and the FN was 0.33. In Figure 8d, one plant was not detected; thus, the TP was 0.83, the FP was 0, and the FN was 0.17. By observing Figure 8, it can be seen that the daytime images offer a more complex problem than the nighttime images, in the sense that the FN and FP were higher in the daytime images. By observing the results, the misdetection or false negative (FN) and false positive (FP) were mainly caused by the illumination changes and shadows—especially the sunlight in the daytime, which produced very bright images taken by the NoIR camera, making it so that the leaf objects were difficult to distinguish from the backgrounds. In a particular condition, the intensity of the leaf was closer to the background, and due to the thresholding technique, it would be considered part of the background; thus, misdetection occurred. In another condition, the shadow caused the non-leaf objects to appear as the leaves, producing a false positive (FP). The results showed that the illumination changes and shadow affected the leaf detection significantly. This led us to propose a method using a sequence of images, as described previously.

#### 3.1.2. Leaf Detection Results Using the NoIR Camera with Static Image Method

**Figure 9:**
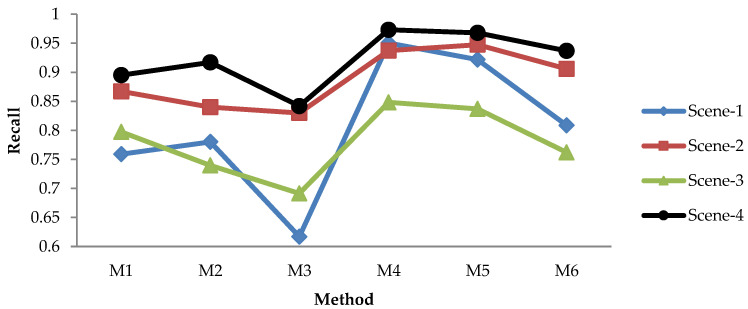
Recall of static image methods from four scenes.

**Figure 10:**
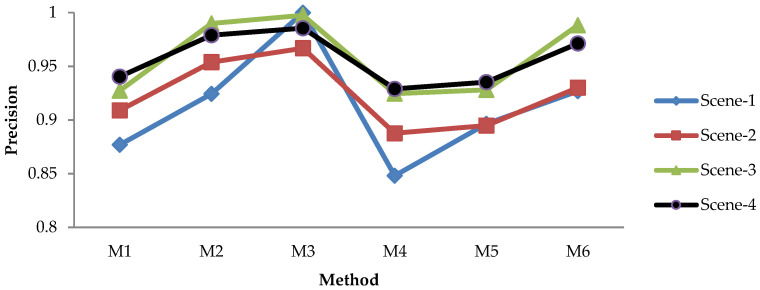
Precision of static image methods from four scenes.

**Figure 11:**
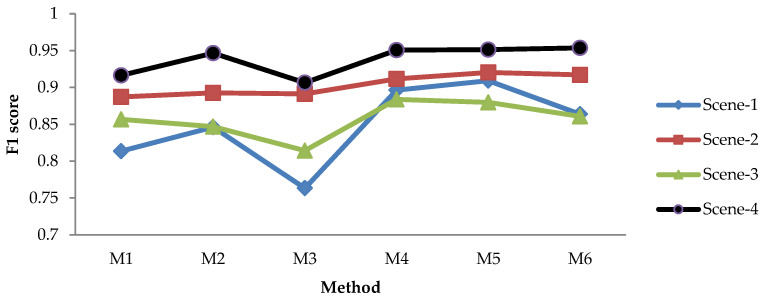
F1 score evaluation of static image methods from four scenes.

**Figure 12:**
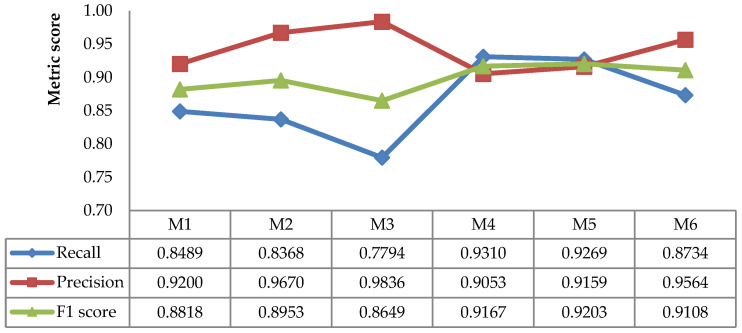
Average recall, precision, and F1 scores of static image methods.

The evaluation results for recall, precision, and F1 score of the static image methods are depicted in Figure 9, Figure 10, Figure 11 and Figure 12. Figure 9 shows the recall of static image methods from four scenes of images. It can be seen from the figures that the combination methods (M4, M5, and M6) achieved a higher recall than the non-combination methods (M1, M2, and M3). Significantly, M4 exhibited the highest value. This result was because of the recall definition given in the previous formula, where the value increased when the TP increased and the FN decreased. From Figure 9, it can be seen that M1 and M2 had the highest recall of the non-combination methods. Therefore, the TP increased by combining them, and the FN decreased; thus, this produced the highest recall. 

Figure 10 shows the precision of static image methods from four scenes of images. The results in Figure 10 show the different patterns from Figure 9: (a) M3 achieved the highest precision, while it had the lowest recall; (b) the precision of the combination methods (M4, M5, and M6) was lower than that of the non-combination methods (M1, M2, M3), while the opposite is shown in Figure 9. The first result was caused by the low TP/FP and high FN produced by M3; therefore, M3 yielded the lowest recall and the highest precision, as shown in Figure 9 and Figure 10, respectively. The second result was caused by the fact that combining the non-combination methods increased both the TP and the FP; thus, it decreased the precision. 

Figure 11 shows the F1 scores of the static image methods from four scenes of images. The figure shows that the combination methods achieved a higher F1 score in each scene than the non-combination methods, similar to the results in Figure 9. It is worth noting that as the F1 score is the harmonic mean of the recall and precision, we may adopt this metric to judge the best method—especially when the recall and precision show a contradictory result.

Figure 9, Figure 10 and Figure 11 show that the effectiveness of the method was affected by the scene. For instance, the recall and F1 score were high for Scene-4 but low for Scene-3. Meanwhile, the precision was high for Scene-4 and Scene-3, and it was low for Scene-1. This suggests that an average value of all scenes should be adopted in order to evaluate the best method more efficiently, as depicted in Figure 12, which provides a comprehensive insight into the performance measurement of the proposed leaf detection method. By observing the figure, we can see that M4 and M5 had the two highest F1 scores, with scores of 0.9167 and 0.9203, respectively. This proves that the proposed combination methods increased the detection performance effectively.

#### 3.1.3. Leaf Detection Results Using NoIR Camera with Image Sequence Method

**Figure 13:**
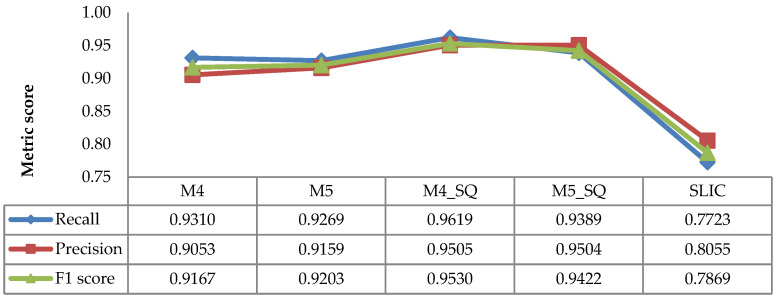
Average recall, precision, and F1 scores of the static image and image sequence methods.

The evaluation results for recall, precision, and F1 scores of the image sequence methods are depicted in Figure 13. The figure shows the static techniques (M4 and M5), the image sequence methods (M4_SQ and M5_SQ), and the pre-existing method (SLIC). As shown in the figure, the recall, precision, and F1 scores of both static image and image sequence methods were superior to those of the SLIC. 

The results show that the strategy of using image sequence works effectively. The image sequence techniques increased the detection performance, as indicated by the improvements in the recall, precision, and F1 scores of the methods (M4_SQ and M5_SQ) compared to their respective static image methods (M4 and M5). The results prove that the TP increases when considering the previous detection in the sequence of images, while the FN decreases. The strategy of dismissing the detection in the daytime images was able to reduce the FP. According to Figure 13, the highest performance was achieved by M4_SQ, with an F1 score of 0.9530.

#### 3.1.4. Results of Execution Time

The evaluation of the execution time of the proposed leaf detection method is given in Table 3. The table shows that the execution time of non-combination methods (M1, M2, and M3) increased according to the number of thresholds. Thus, the execution of M1 (one threshold) was the lowest (275.76 ms), while that of M3 (three thresholds) was the highest (1247.63 ms). Meanwhile, the execution times of the combination methods (M4, M5, and M6) were the sum of those of the non-combinational methods. It is worth noting that the proposed image sequence methods reduced the execution time from their respective static image methods, i.e., from 551.00 ms to 516.30 ms (M4_SQ), and from 1500.15 ms to 1408.07 ms (M5_SQ). 

Recalling the previous results in terms of the F1 score and execution time, we may conclude that M4_SQ is the best leaf detection method.

#### 3.1.5. Leaf Detection Results Using Benchmark Image Datasets

**Figure 14:**
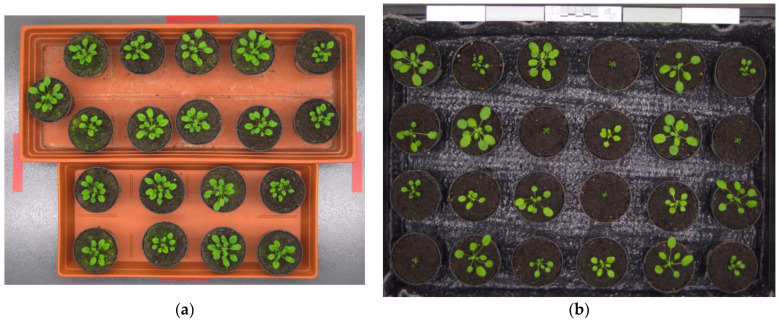
Image datasets of *Arabidopsis thaliana* plants [12,39,40]: (**a**) Ara2012; (**b**) Ara2013.

As described previously, one of the main contributions of our proposed system is an approach to combine the bi-level and multilevel Otsu thresholding techniques to detect leaves. The experimental results discussed in Section 3.1.1, Section 3.1.2, Section 3.1.3 and Section 3.1.4 prove that this approach worked effectively for the images taken using an NoIR camera. In this work, we extended the evaluation of our proposed combination method using the benchmark image datasets from [12,39,40]. We selected two datasets that were suitable for the leaf detection, i.e., the images of *Arabidopsis thaliana* plants on the tray—namely, Ara2012 and Ara2013, as illustrated in Figure 14a,b, respectively.

The images of Ara2012 and Ara2013 were taken using a 7-megapixel Canon camera, with a resolution of 3108 × 2324 pixels. The images were taken during the daytime, every 6 h, over 21 days for Ara2012, and every 20 min over 49 days for Ara2013. The image acquisition was conducted in the laboratory, using artificial lighting to emulate the daylight. Ara2012 consisted of 16 images, where each image contained 19 plants, as shown in Figure 14a. Ara2013 consisted of 27 images, where each image contained 24 plants, as shown in Figure 14b.

**Figure 15:**
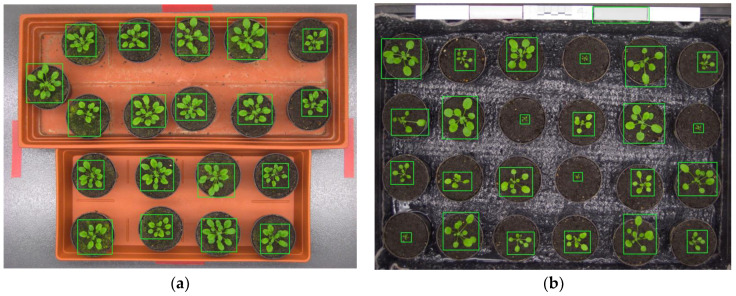
Typical leaf detection results of *Arabidopsis thaliana* plants: (**a**) Ara2012; (**b**) Ara2013.

Since our proposed algorithm was intended for infrared images, we could not adopt our algorithm directly to Ara2012 and Ara2013, because the datasets were visible images. Fortunately, we can implement the approach of combining bi-level and multilevel Otsu thresholding to those datasets. Thus, instead of using the grayscale images described in Section 3.2, we modified our algorithm to use the “a” channel of the Lab color space, as proposed in [12]. Typical leaf detection results are illustrated in Figure 15a for Ara2012 and Figure 15b for Ara2013. All plants were detected successfully in both figures, as shown by the bounding boxes of detected leaves in the images; however, a false positive detection occurred in Figure 15b.

**Figure 16:**
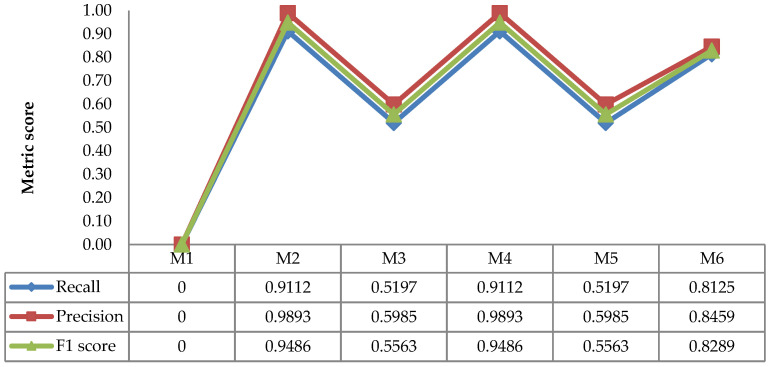
Average recall, precision, and F1 scores of Ara2012.

**Figure 17:**
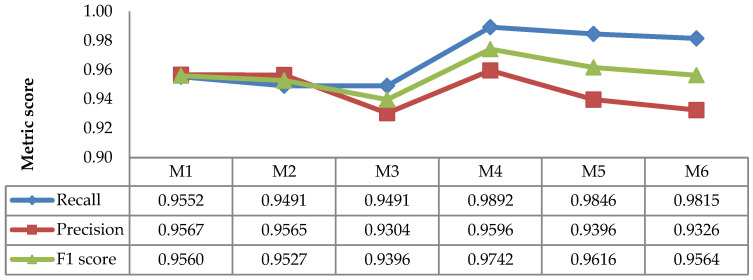
Average recall, precision, and F1 scores of Ara2013.

The evaluation results of the recall, precision, and F1 scores of Ara2012 and Ara2013 using M1 to M6 are shown in Figure 16 and Figure 17, respectively. It should be noted that M1 could not detect leaves in Ara2012; this was caused by the fact that the images of Ara2012 were composed of three distinctively colored objects (leaf, soil, and tray). Thus, bi-level thresholding (M1) failed to separate leaves from the background. Meanwhile, even though Ara2013 was composed of the same three objects, their color was not remarkably different; therefore, bi-level thresholding (M1) could be used to extract the leaves.

The results in Figure 16 and Figure 17 show that the multilevel Otsu thresholding achieved the highest F1 score in both Ara2012 and Ara2013. Moreover, it is worthy of note that the proposed combination method (M4) achieved the highest F1 score. 

Since the existing works that used the Ara2012 and Ara2013 images measured the FBD to evaluate the performance of the leaf detection (segmentation) algorithm, we computed the FBD for a fair comparison. The comparison results are given in Table 4, where the FBD is given as the mean and standard deviation (in parentheses). The result show that the FBD of our proposed method achieved high values of 93.7% and 96.2% for Ara2012 and Ara2013, respectively. These values were close to those of the existing methods.

### 3.2. Leaf Counting Results

#### 3.2.1. Leaf Counting Results Using the NoIR Camera

**Figure 18:**
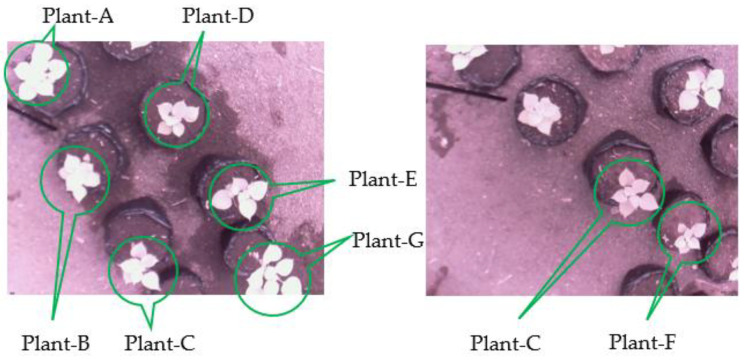
Position of Plant-A to Plant-G in the leaf counting experiments.

As discussed in the previous section, four scenes of images were used in the experiments. Since the position of a plant differed in each scene, we categorized the plants as seven plants—namely, Plant-A to Plant-G—as depicted in Figure 18. The relationships between the scene, the plant, and the number of leaves (ground truth) are given in Table 5. Then, these seven plants were used to evaluate the performance of the leaf counting algorithm, as discussed below. Since the leaf counting was conducted after the leaf detection, we selected the two best leaf detection methods—i.e., M4_SQ and M5_SQ—to evaluate the performance of the leaf counting algorithm. The algorithm’s performance was measured using the difference in count (DiC), absolute difference in count (ABS_DIC), and execution time. To observe the effects of day and night images, we compared the performance of the lead counting according to them.

**Figure 19:**
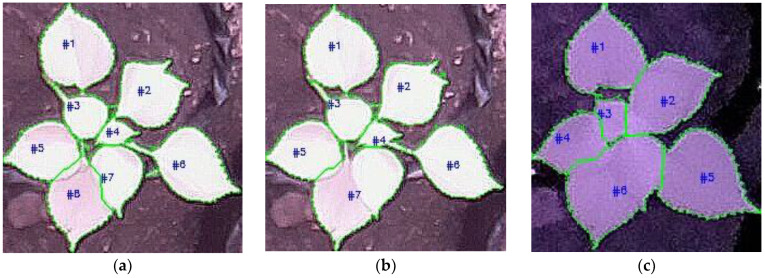

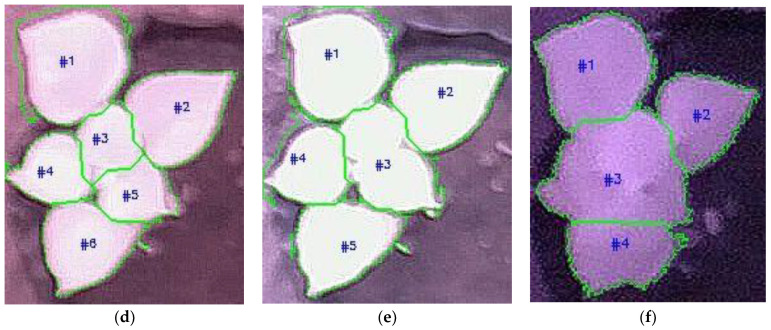
Leaf counting results using NoIR camera: (**a**,**d**) without miscounting errors; (**b**,**c**,**e**,**f**) with miscounting errors.

The samples of leaf counting results are depicted in Figure 19, where the detected leaves are bounded with green lines and numbered. In the figure, the images in the first row are of Plant-C in Scene-3. Meanwhile, the images in the second row are of Plant-C in Scene-2. Figure 19a,b,d,e are the daytime images, while Figure 19c,f are the nighttime images. Figure 19a shows the leaf counting result in which all leaves were detected and counted successfully. Figure 19b,c show the counting results of the same plant as in Figure 19a; however, one leaf and two leaves were miscounted in Figure 19b,c, respectively. Similar results are shown in Figure 19d–f, where all leaves were counted properly in Figure 19d, but one leaf and two leaves were miscounted in Figure 19e,f, respectively.

**Figure 20:**
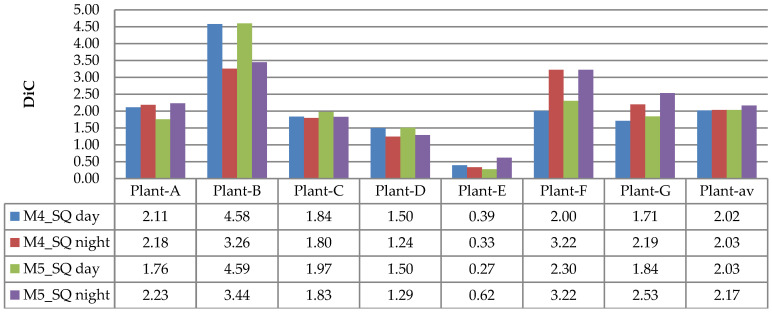
Results of DiC measurements using the NoIR camera.

**Figure 21:**
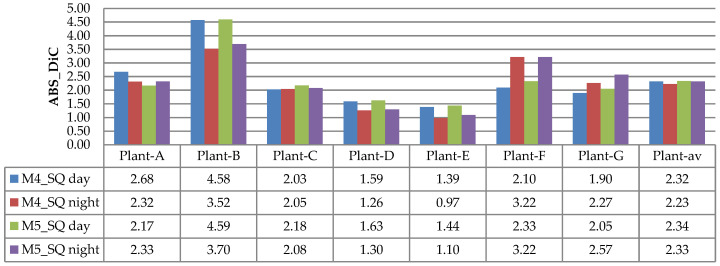
Results of ABS_DiC measurements using the NoIR camera.

The evaluation results of DiC and ABS_DiC are given in Figure 20 and Figure 21, respectively. In the figures, the performances of leaf counting in the day and night images are compared using seven plants: Plant-A to Plant-G. The Plant-av represents the average value of the seven plants. 

Figure 20 and Figure 21 show that both DiC and ABS_DiC had a similar characteristic, where the lowest value (best performance) was achieved for Plant-E (i.e., DiC of 0.27 for M5_SQ day, and ABS_DiC of 0.97 for M4_SQ night), while the highest value (lowest performance) was from Plant-B (i.e., DiC of 4.59 for M5_SQ day, and ABS_DiC of 4.59 for M5_SQ day). Too many overlapping leaves caused miscounting in Plant-B—these leaves failed to be separated by the algorithm. The figures show that both day and night images had similar results, in the sense that in some plants the day images achieved better results, but in other plants achieved worse results. The values of Plant-av were almost identical between the day and night images. These results are consistent with the observation of the images in Figure 19.

To evaluate the execution time of the leaf counting algorithms, the time taken to count the leaves per plant was determined. From the experiments, the average execution time was 545.41 ms, which was fast enough for this application.

#### 3.2.2. Leaf Counting Results Using Benchmark Image Datasets

**Figure 22:**
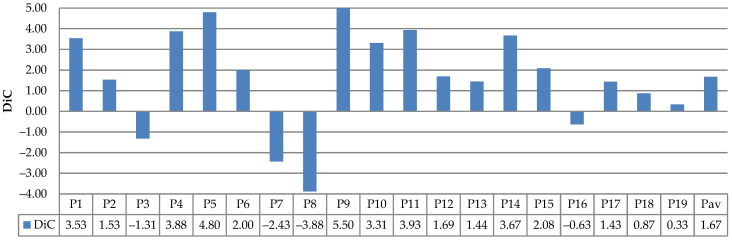
Results of DiC measurements using Ara2012.

**Figure 23:**
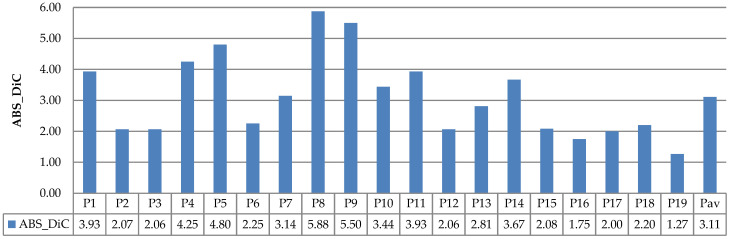
Results of ABS_DiC measurements using Ara2012.

**Figure 24:**
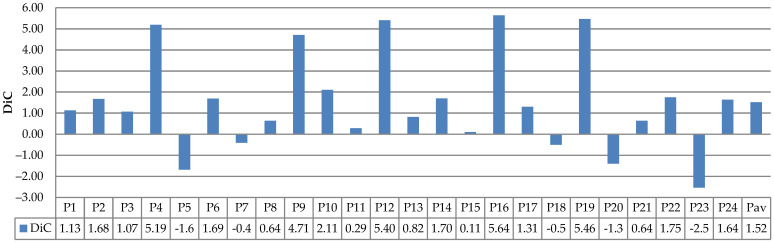
Results of DiC measurements using Ara2013.

**Figure 25:**
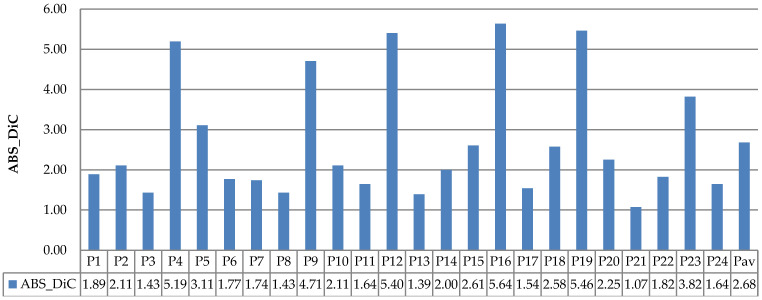
Results of ABS_DiC measurements using Ara2013.

The evaluation results of leaf counting using the existing datasets (Ara2012 and Ara2013) are depicted in Figure 22, Figure 23, Figure 24 and Figure 25. Figure 22 and Figure 23 show the DiC and ABS_DiC of Ara2012, respectively. Figure 24 and Figure 25 show the DIC and ABS_DiC of Ara2013, respectively. Pn represents the number of *Arabidopsis* plants in the figures, where the numerical order is from left to right and from top to bottom of Figure 14 and Figure 15. Pav is the average value of the plants.

Figure 22 and Figure 23 show that lower values of DiC and ABS_DiC of Ara2012 were achieved by the plants with a few non-overlapping leaves (such as Plant3, Plant16, Plant18, and Plant19). The plants with overlapping leaves (Plant4, Plant5, Plant8, and Plant9) showed higher DiC and ABS_DiC.

The performance of leaf counting of Ara2013 shown in Figure 24 and Figure 25 was affected by the overlapping leaves and the size of the leaves. The small leaves (Plant 4, Plant9, Plant12, Plant16, and Plant19) achieved higher DiC and ABS_DiC. Plant23 had a high DiC because of its overlapping leaves. Similarly to Ara2012, the plants with a few overlapping leaves achieved lower values of DiC and ABS_DiC. 

To establish the feasibility of the values of DiC and ABS_DiC, we compared them with the existing works given in Table 6. The values in the table are expressed as the mean and standard deviation (in parentheses). From the table, we may conclude that the performance of our proposed leaf counting method was within reasonable values compared to the existing works; in particular, for both datasets (Ara2012 and Ara2013), the DiC and ABS_DiC of our proposed method were within the average values of the existing techniques. Moreover, the DiC and ABS_DiC of our proposed method using the NoIR camera were slightly lower than those of Ara2012 and Ara2013. This implies that our proposed leaf counting method is a feasible technique. Fortunately, our proposed method did not need many prepared data samples for the training process, and was suitable for a real-time system.

## 4. Conclusions

A leaf detection and counting method using a low-cost infrared camera system was developed. The image dataset contained *Ramie* plant leaves captured during the day and nighttime in outdoor environments. The datasets provided comprehensive images under illumination changes, low contrast, and shadows. The proposed method took the benefit of the infrared imaging, allowing the Otsu thresholding to work effectively. The combination of single- and multilevel thresholds was developed to deal with illumination changes and shadow problems. Both static images and image sequences were evaluated, where the image sequence method showed superiority over the static method. The leaf counting method adopted the watershed algorithm for separating the leaves. The proposed leaf detection method achieved a high performance, as indicated by the high F1 score of 0.9530 for the image sequence approach. The performance of the proposed leaf counting method measured using the difference in count (DiC) was 2.02. Furthermore, the proposed leaf detection and leaf counting methods were evaluated using the benchmark image datasets, and achieved feasible values; thus, they are comparable with the existing techniques. Moreover, the execution time of the proposed algorithm was approximately one second, which is suitable for a real-time leaf monitoring system.

In the future, we will improve and extend the algorithms to cope with more complex backgrounds. Furthermore, we will investigate the implementation of our approach in natural environments. 

## Figures and Tables

**Table 1:** Existing leaf detection and counting techniques.

Ref.	Algorithm	Implementation	Type of Images	Lighting Condition of Images	Purpose
[3]	CIELAB color thresholding	PC—MATLAB	Visible images	Indoor	LD
[4]	HSI color segmentation	PC—C++	Visible images	Indoor	LD
[5]	GrabCut	PC—OpenCV	Visible images	Indoor	LD
[6]	New channel color segmentation	Raspberry Pi—OpenCV	Visible and NoIR images	Outdoor	LD
[7]	Random walker	PC—MATLAB	Visible images	Outdoor	LC
[8]	Shape-based segmentation	PC—NA	Visible images	Outdoor	LD
[9]	Contour edges detection	PC—OpenCV	Visible images	Outdoor	LD
[10]	Active snake model	PC—MATLAB	Visible images	Outdoor	LD
[11]	Graph, CHT	PC—MATLAB	Visible image	Outdoor	LD, LC
[12]	3D histogram, SLIC, watershed	PC—MATLAB	Visible images	Indoor	LD, LC
[13]	Random walker	PC—NA	Visible images	Outdoor	LD
[14]	HSV color thresholding	PC—NA	Visible images	Outdoor	LD
[15]	CIELAB color thresholding	PC—MATLAB	Visible images	Outdoor	LD
[16]	Watershed, GrabCut	PC—NA	Visible images	Outdoor	LD
[17]	Watershed	PC—MATLAB	Visible images	Indoor (with window)	LC
[18]	Watershed	PC—MATLAB	Visible images	Indoor	LC
[19]	Expectation-maximization	PC—MATLAB	Visible image	Outdoor	LD
[20]	Mask R-CNN	PC—NA	Visible images	Outdoor	LD
[21]	MLP-ASM	PC—MATLAB	Visible images	Outdoor	LD
[22]	Orthogonal transform, DCNN	PC—MATLAB	Visible images	Outdoor	LD, LC
[23]	DNN	PC—Keras	Visible, NIR, fluorescent images	Outdoor	LC
[24]	DNN	PC, Android device—OpenCV	Visible images	Indoor	LC
[25]	NN, watershed	PC—NA	Visible images	Outdoor	LD, LC
[26]	DNN	PC—NA	Visible images	Outdoor	LC

**Table 2:** Proposed leaf detection techniques.

Method	Description
M1	Bi-level Otsu thresholding (Single threshold)
M2	Three-level Otsu thresholding (Two thresholds)
M3	Four-level Otsu thresholding (Three thresholds)
M4	Bi-level + three-level Otsu thresholding (M1 + M2)
M5	Bi-level + four-level Otsu thresholding (M1 + M3)
M6	Three-level + four-level Otsu thresholding (M2 + M3)
M4_SQ	M4 with the sequence of images
M5_SQ	M5 with the sequence of images
SLIC	SLIC method proposed by [12]

**Table 3:** Execution time of leaf detection algorithm.

Method	Execution Time (ms)
M1	275.76
M2	302.79
M3	1247.63
M4	551.00
M5	1500.15
M6	1498.92
M4_SQ	516.30
M5_SQ	1408.07
SLIC	16,116.11

**Table 4:** Comparison of leaf detection performance.

Method (Ref.)	FBD * (%)	
	Ara2012	Ara2013
[11]	96.2 (1.9)	96.2 (2.4)
[12]-IPK	97.0 (0.8)	96.3 (1.7)
[12]-Nottingham	95.3 (1.1)	93.0 (4.2)
[12]-MSU	94.0 (1.9)	87.7 (3.6)
[12]-Wageningen	94.7 (1.5)	95.1 (2.0)
[22]	95.5 (2.3)	96.3 (2.4)
Proposed	93.7 (2.0)	96.2 (1.7)

*: mean (standard deviation).

**Table 5:** Relationships between the scene, plant, and number of leaves.

	Number of Leaves						
Scene	Plant-A	Plant-B	Plant-C	Plant-D	Plant-E	Plant-F	Plant-G
Scene-1	5	8	6	NA *	NA *	NA *	NA *
Scene-2	6	8	7	6	6	NA *	NA *
Scene-3	6	8	8	NA *	6	6	NA *
Scene-4	8	8	8	7	6	NA *	7

NA * = Plant does not appear in the scene.

**Table 6:** Comparison of the values of DiC and ABS_DiC.

Method (Ref.)	Ara2012		Ara2013		NoIR	
	DiC *	ABS_DiC *	DiC *	ABS_DiC *	DiC *	ABS_DiC *
[11]	−0.9 (2.5)	2.0 (1.8)	1.2 (5.9)	3.8 (4.7)	NA	NA
[12]-IPK	−1.8 (1.8)	2.2 (1.3)	−1.0 (1.5)	1.2 (1.3)	NA	NA
[12]-Nottingham	−3.5 (2.4)	3.8 (1.9)	−1.9 (1.7)	1.9 (1.7)	NA	NA
[12]-MSU	−2.5 (1.5)	2.5 (1.5)	−2.0 (1.5)	2.0 (1.5)	NA	NA
[12]-Wageningen	1.3 (2.4)	2.2 (1.6)	−0.2 (0.7)	0.4 (0.5)	NA	NA
[22]	0.12 (0.78)	0.55 (0.56)	−0.22 (1.56)	1.11 (1.05)	NA	NA
[23]	−0.39 (1.17)	0.88 (0.86)	−0.78 (1.64)	1.44 (1.01)	NA	NA
Proposed	1.67 (2.46)	3.11 (1.33)	1.52 (2.29)	2.68 (1.49)	2.02 (1.27)	2.23 (0.93)

*: Mean (standard deviation).

## Data Availability

Not applicable.
